# A Small-Displacement Sensor Using Total Internal Reflection Theory and Surface Plasmon Resonance Technology for Heterodyne Interferometry

**DOI:** 10.3390/s90402498

**Published:** 2009-04-14

**Authors:** Shinn-Fwu Wang

**Affiliations:** Department of Electronic Engineering, Ching Yun University, No.229, Chien-Hsin Rd., Jhongli City, Taoyuan, 320, Taiwan, ROC; E-Mail: sfwang@cyu.edu.tw; Tel.: +886-3-4581196, ext. 5113; Fax: +886-3-4588924

**Keywords:** Total internal reflection (TIR), surface plasmon resonance (SPR), heterodyne interferometry (HI), small-displacement sensor

## Abstract

A small-displacement sensor based on total-internal reflection theory and surface plasmon resonance technology is proposed for use in heterodyne interferometry. A small displacement can be obtained simply by measuring the variation in phase difference between s- and p-polarization states with the small-displacement sensor. The theoretical displacement resolution of the small-displacement sensor can reach 0.45 nm. The sensor has some additional advantages, e.g., a simple optical setup, high resolution, high sensitivity and rapid measurement. Its feasibility is also demonstrated.

## 1. Introduction

As is well known, small displacement measurement plays an important role in high technology industries, especially in the liquid crystal display (LCD) manufacturing process and semiconductor production. Over the past few decades several articles have proposed ways of increasing the resolution of small displacement measurements [1–4]. In 2001, Nesci *et al*. proposed a coherent photon scanning tunneling microscope [1] with a resolution of 1.6 nm. The setup employs heterodyne interferometry, allowing both the phase and the amplitude of the optical near field to be measured. Liu *et al*. presented a homodyne polarization laser interferometer [2] for high-speed measurement of small displacements. The interferometer had a general measurement resolution of about 0.5 nm. Liao *et al*. proposed a method for small displacement measurement based on the critical angle method and the confocal technique in 2005 [3]. The best resolution can reach at least 5 nm. In addition, Chiu *et al*. presented a method for small absolute distance measurement with nanometer resolution using geometrical optics principles and a SPR angular sensor [4]. They obtained different measurement resolutions by using lens objectives with different numerical apertures.

The methods for measuring small displacement of the research papers [3,4] mentioned above are either based on the Total Internal Reflection (TIR) theory or Surface Plasmon Resonance (SPR) technology. In this paper, a small-displacement sensor by the simultaneous use of the TIR theory and SPR technology is proposed. The small-displacement sensor is made of a right-angle prism with a refractive index of 1.51509 at the wavelength *λ* = 632.8 nm. It should be noted that a right-angle side of the prism isn’t coated with any metal film, but the other side is coated with two metal film layers.

As a heterodyne light source focuses on a mirror driven by a piezoelectric transducer (PZT), the reflected light passes through an objective lens and is incident on a beam-splitter. Afterwards its reflected light from the beam-splitter is refracted into the hypotenuse of the right-angle prism. At first, the light is incident on a right-angle side of the prism that not metal coated. Next, the reflected light is incident on the other side that is coated with two metal layers. Finally, the light is detected by a linear photo-detector when it passes through the hypotenuse of the right-angle prism and an analyzer. As the mirror departs from the focal plane, the beam converges or diverges into the prism. The two marginal rays of the beam exiting the prism will induce different phase difference variations between the s- and p-polarizations. Some special equations are derived according to the optical configuration and Fresnel’s equations [5]. By substituting the phase difference data into these equations, small displacements can be measured. The method has some practical advantages: e.g., a simple optical setup, high resolution, high sensitivity and rapid measurement. In addition its feasibility is demonstrated.

## 2. Principle

### 2.1. The phase difference at the TIR effect

**Figure 1.**
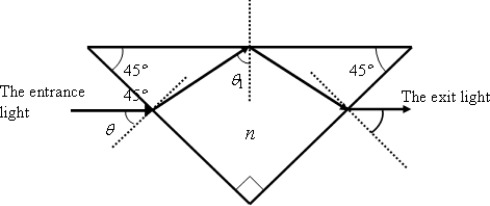
A ray of light in air incident at *θ* on one side surface of a right-angle prism with refractive index *n*.

A ray of light in air is incident with an angle *θ* on one side of a right-angle prism with refractive index *n*, as shown in Figure 1.

The light ray is refracted into the prism and it propagates toward the hypotenuse surface of the prism. At that surface, there is a boundary between the prism and air. If the angle of incidence at the boundary is *θ*_1_, then we have:

(1) $$\theta_{1}=45^{{}^{\circ}}+{\text{sin}}^{-1}\left(\frac{\text{sin}\;\theta }{n}\right)$$

Here the signs of *θ*_1_ and *θ* are defined as positive if they are measured clockwise from a surface normal. If *θ*_1_ is larger than the critical angle *θ*_C_, the light is totally reflected at the boundary. According to Fresnel’s equations, the phase difference between s- and p-polarizations is given as:

(2) ϕ1=2 tan−1{sin2[45°+sin−1(sin θn)]−1n2tan[45°+sin−1(sin θn)] sin[45°+sin−1(sin θn)]}.

From Equation (2), the variation *Δθ* of the incident angle can be written as:

(3) Δθ≅(n2 tan2θ1−1)(n2 sin2θ1−1)122n sin θ1[2−(n2−1) tan2 θ1]×(n2−sin2 θ)12cos θΔϕ1=A(θ)Δϕ1

where *Δθ*_1_ is the phase difference variation and:

(4) A(θ)=(n2 tan2 θ1−1)(n2 sin2 θ1−1)122n sin θ1[2−(n2−1)tan2 θ1]×(n2−sin2 θ)12cos θ..

### 2.2. The basic principle of SPR technology

**Figure 2.**
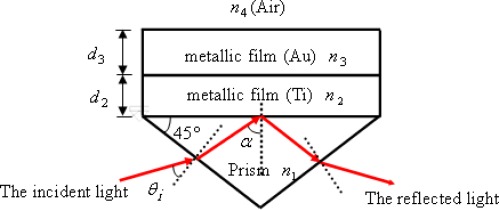
Kretchmann’s configuration for the generation of SPR.

In this paper, a right-angle prism with a four-layer device [prism-titanium(Ti)-gold(Au)-air] in the Kretchmann’s configuration [6] is used. For the Kretchmann configuration of the four-layer system as shown in Figure 2, the surface plasmons are excited when *α* equals to the resonant angle *α_sp_*.

From Maxwell’s equations, the reflection coefficients of p- and s-polarizations can be expressed as [7]:

(5) $$r_{1234}^{t}=\frac{r_{12}^{t}+r_{234}^{t}e^{i2k_{z2}d_{2}}}{1+r_{12}^{t}r_{234}^{t}e^{i2k_{z2}d_{2}}},$$

(6) $$r_{234}^{t}=\frac{r_{23}^{t}+r_{34}^{t}e^{i2k_{z3}d_{3}}}{1+r_{23}^{t}r_{34}^{t}e^{i2k_{z3}d_{3}}}$$

where $r_{\mathrm{ij}}^{t}=\frac{E_{i}^{t}-E_{j}^{t}}{E_{i}^{t}+E_{j}^{t}}$, *d*_2_ and *d*_3_ are the thicknesses of medium 2 and medium 3, respectively, and t = p, s,

(7) $$E_{I}^{t}=\{\begin{array}{cc} n_{I}^{2}/k_{zI} & t=p\\ k_{zI} & t=s \end{array},\;\;I=i,\;j;i,\;j=1,2,3,4.$$

In Equation (7)*k_zi_*_(_*_j_*_)_ is the component of the wave vector in a medium *i*(*j*) in the *z* direction and is given as:

(8) $$k_{\mathrm{zi}(j)}=k_{0}\;(n_{i(j)}^{2}-n_{1}^{2}\;{\text{sin}}^{2}\;\alpha ),$$

where *n*_1_ is the refractive index of the prism, *n*_2_ is the refractive index of Ti metal, *n*_3_ is the refractive index of Au metal, *n*_4_ is the refractive index of air and *k*_0_ is the wave vector in vacuum. If the amplitude reflection coefficients $r_{1234}^{p}$ and $r_{1234}^{s}$ are written as:

(9) $$r_{1234}^{p}=\left|r_{1234}^{p}\right|e^{{i\phi }_{p}},r_{1234}^{s}=\left|r_{1234}^{s}\right|e^{{i\phi }_{s}},$$

then the phase difference *ϕ* between p- and s- polarization components is:

(10) $$\phi =\phi_{p}-\phi_{s}$$

Besides, the reflectivities of p- and s- polarization components are $R_{p}={\left|r_{1234}^{p}\right|}^{2}$ and $R_{s}={\left|r_{1234}^{s}\right|}^{2}$, respectively.

Because the phase difference variation *Δϕ*_2_ due to the SPR effect is a function of the rotation angle or deviation angle *Δθ* [8], the phase difference variation can be given by:

(11) $$\Delta \phi_{2}=B(\theta )\Delta \theta$$

where *B*(*θ*) is a parameter dependent on the initial incident angle.

### 2.3. The basic principle of the displacement probe (DP)

**Figure 3.**
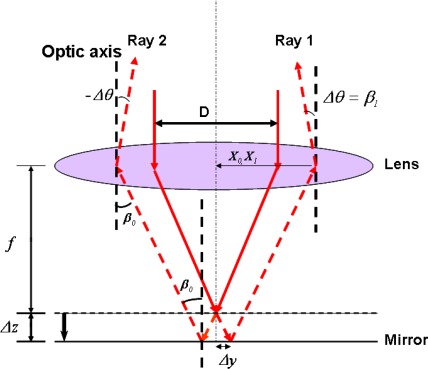
Rays back from the DP (*f*: the focal length of an objective lens, *D*: the diameter of the beam, *Δθ*: the angular deviation from optic-axis, *β_0_*: the sloping angle of the ray incident on the mirror, *Δz*: the displacement of the mirror, *Δy*: the distance off the optical axis if *Δz* exits)

Figure 3 shows the structure of a DP; it consists of an objective lens with a focal length *f* and a mirror located near the focal plane of an objective lens. A light ray coming from a heterodyne light source is incident on the objective lens and is reflected by the mirror. The reflected light passes through the objective lens again. And it is in the anti-parallel direction with the incident beam if the mirror is just located at the focal plane. Otherwise it will have a deviation angle *Δθ* with the anti-direction of the incident beam. From the refraction ray- tracing equation, we can achieve:

(12) $$\Delta z≅-\frac{f^{2}}{D}\Delta \theta$$

From Equation (12), it can be seen that the displacement *Δz* is almost proportional to *Δθ*.

**Figure 4.**
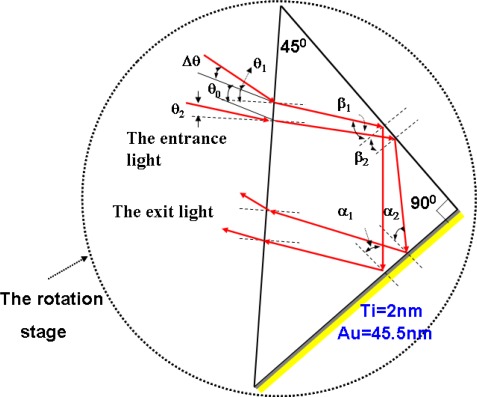
The incident angles of the two marginal rays that lie on the plane perpendicular to the hypotenuse of the right-angle prism are *θ*_1_ = *θ*_0_ + *Δθ* and *θ*_2_ = *θ*_0_ − *Δθ*, respectively.

Figure 4 shows the scheme of the small-displacement sensor. The small-displacement sensor is made of a right-angle prism with a refractive index of 1.51509 at the wavelength *λ* = 632.8 nm. A right-angle side of the right-angle prism isn’t coated with any metal film, but the other side is coated with two layers of metal film. If the light reflects from the DP (see Figure 3), it later enters the small-displacement sensor.

Suppose that the defocusing amount *Δz* exists, the reflected light coming from the DP is convergent or divergent. Suppose the reflected light is divergent, the incident angle on the hypotenuse of the right-angle prism is not unique. In Figure 4, the incident angles of the two marginal rays that lie on the plane perpendicular to the hypotenuse of the right-angle prism are *θ*_1_ = *θ*_0_ + *Δθ* and *θ*_2_ = *θ*_0_ − *Δθ*, respectively, where *θ*_0_ is the initial incident angle as *Δz* = 0. It must be noted that the initial incident angle *θ*_0_ is chosen when the incident angle *α* equals to the resonant angle *α_sp_*, i.e. *θ*_0_ = sin^−1^[*n*sin(*α_sp_*−45°)]. This is because that the SPR effect has a very steep phase change around the resonant angle [8].

We can then obtain the incident angles of the two marginal rays that are incident at one side of the right-angle prism (the surface of the side is uncoated metal) are $\beta_{1}=45{}^{\circ}+{\text{sin}}^{-1}[\frac{\text{sin}(\theta_{0}+\Delta \theta )}{n}]$ and $\beta_{2}=45{}^{\circ}+{\text{sin}}^{-1}[\frac{\text{sin}(\theta_{0}-\Delta \theta )}{n}]$, respectively. If the phase difference variation is *Δϕ*_1_ for one marginal ray indent on the side of the right-angle prism at the incident angle *β*_1_, then we can obtain the phase difference variation *Δϕ*_1_^′^ = −*Δϕ*_1_ for the other marginal ray at the incident angle *β*_2_. Thus we can achieve the phase difference variation on account of the TIR effect is:

(13) $$\Delta \phi_{t1}=\Delta \phi_{1}-\Delta \phi_{1}{}^{\prime }\approx 2\Delta \phi_{1}.$$

Combining Equation (3) with (13), we have:

(14) $${\Delta \phi }_{t1}\approx 2\frac{\Delta \theta }{A(\theta )}$$

Similarly, the incidence angles of the two marginal rays that are incident at the other side (coated with two metal layers) are $\alpha_{1}=45{}^{\circ}-{\text{sin}}^{-1}[\frac{\text{sin}(\theta_{0}+\Delta \theta )}{n}]$ and $\beta_{2}=45{}^{\circ}-{\text{sin}}^{-1}[\frac{\text{sin}(\theta_{0}-\Delta \theta )}{n}]$, respectively.

If the phase difference variation is *Δϕ*_2_ for one marginal ray incident on the side of the right-angle prism at the incidence angle *α*_1_, then we can obtain the phase difference variation *Δϕ*_2_^′^ = −*Δϕ*_2_ for the other marginal ray at the incidence angle *α*_2_. Thus we can achieve the phase difference variation on account of the SPR phenomenon as:

(15) $${\Delta \phi }_{t2}={\Delta \phi }_{2}-{\Delta \phi }_{2}{}^{\prime }\approx {2\Delta \phi }_{2}.$$

From Eqs (11) and (15), *Δϕ_t_*_2_ can be expressed by:

(16) $${\Delta \phi }_{t2}\approx 2B(\theta )\Delta \theta$$

Therefore, the total phase difference variation *Δϕ_t_* is given as:

(17) $${\Delta \phi }_{t}={\Delta \phi }_{t1}+{\Delta \phi }_{t2}\approx 2(\frac{1}{A(\theta )}+B(\theta ))\cdot \Delta \theta =C(\theta )\cdot \Delta \theta$$

where $C(\theta )=2(\frac{1}{A(\theta )}+B(\theta ))$. According to Eqs. (12) and (17), the total phase difference variation *Δϕ*_2_ due to the displacement *Δz* can be written as:

(18) $${\Delta \phi }_{t}\approx -C(\theta )\cdot \frac{D}{f^{2}}\cdot \Delta z.$$

## 3. Experimental Apparatus and Results

**Figure 5.**
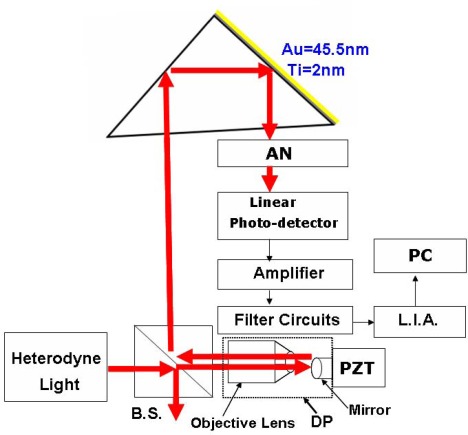
The experimental configuration (AN: analyzer; PZT: piezoelectric transducer, LIA: lock-in amplifier; BS: beam splitters, PC: personal computer).

Figure 5 shows the experimental configuration that is used for measuring small displacements by TIR and ATR in heterodyne interferometry. In the experiment, the numerical aperture, N.A., of the objective lens (*D* = 4.93 mm and *f* = 2.9 mm) is 0.85. In Figure 1, the parameters of the four-layer device (BK7 glass prism-Ti-Au-air) are the Ti film thickness *d*_2_ of 2 nm, the Au film thickness *d*_3_ of 45.5 nm, and the wavelength λ of 632.8 nm. The permittivities of Bk-7 glass prism (*ε*_1_ = *n*_1_^2^), Ti film (*ε*_2_ = *n*_2_^2^), Au metal (*ε*_2_ = *n*_3_^2^), and air (*ε*_4_ = *n*_4_^2^) are *ε*_1_ = (1.51509)^2^, *ε*_2_ = −3.84+12.5*i*, *ε*_3_ = −12+1.26*i* and *ε*_4_ = (1.0003)^2^, respectively.

**Figure 6.**
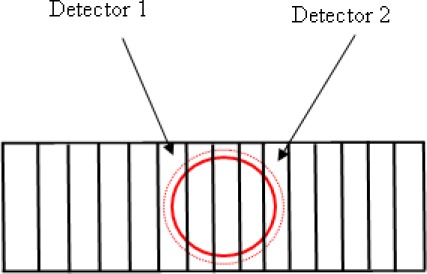
Two marginal rays passing through an analyzer detected by a linear photo-detector.

As shown in Figure 5, a heterodyne light source [9] travels through the beam splitter BS and enters the DP. Initially, a heterodyne light source focuses on a mirror that is driven by a PZT with a closed-loop resolution of 0.3 nm (PI, model P-621.ZCD), the reflected light passes through an objective lens and is incident on a beam-splitter. After that its reflected light from the beam-splitter is refracted into the hypotenuse of a right-angle prism. Afterwards, the light is incident on a side of the right-angle prism (the surface of that side is uncoated with metal). Then the reflected light is incident on the other side that is coated with two layers of metal. Finally, the light is detected by a linear photo-detector (see Figure 6, A5V-38, UDT Sensors, Inc.) when it passes through the hypotenuse of the right-angle prism and an analyzer AN. Thus, the small displacements can be measured by measuring the total phase difference variation that is achieved by a lock-in amplifier (SR830; Stanford Research Systems, Sunnyvale, CA, USA) with an angular resolution of 0.01°.

In addition, I must design a band-pass filter in order to filter out the high and low frequency noises. Owing to the use of the beat frequency of 2-KHz in the experimental setup, this band-pass filter can be easily designed. For the sake of convenience, the band-pass filter was designed using a resister tunable filter (model: RT-3BP1/2, manufactured by NF Corporation) and some electronic components.

**Figure 7.**
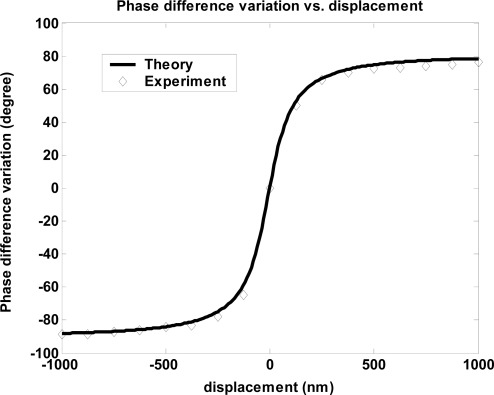
The experimental and theoretical curves of the total phase difference variation versus the displacement *Δz*.

The experimental and theoretical curves of the total phase difference variation *Δϕ_t_* versus the displacement *Δz* are shown in Figure 7. In the experiment, the phase difference variation is obtained as the displacement varies from *Δz =* −1,000 nm to *Δz =* 1,000 nm in one sense. It is clear that the experimental results and the theoretical curve are in good agreement. The measurement range of the displacement *Δz* is thus −1,000 nm ≤ *Δz* ≤ +1,000 nm.

## 4. Discussion

**Figure 8.**
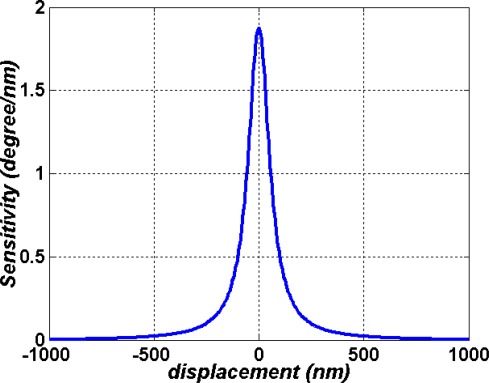
The sensitivity *S* versus displacement.

At this moment, let me discuss the sensitivity of the small-displacement sensor of small displacement measurement by use of multiple internal reflections in heterodyne interferometry. The sensitivity *S* of the system is defined as:

(19) $$S=\frac{d(\delta \phi )}{\mathrm{dz}}$$

where *δϕ* is the phase difference variation and *dz* is a small-displacement change made by PZT. As shown in Figure 8, we can obtain the curve of sensitivity *S* versus *θ*. It is clear that the sensitivity can reach 1.8 (degree/nm).

In this article, a heterodyne interferometer is used to measure a small displacement. Owing to its common-path configuration, it has the advantages of high resolution, high stability and real-time measurement. It also has some inherent nonlinear errors due to imperfect optical components, misalignment, or operating defects that will reduce the system performance. Generally speaking, the inherent nonlinear errors accompanying the phase differences during the phase range of 0 ∼ 2*π* are periodic errors. They can be classified to two kinds, i.e., the first order error and the second harmonic error.

### (a). The First Order Error

The first order error accompanying the phase range 0 ∼ 2*π* has the same cycle. The errors are frequency mixing error and polarization mixing error due to:

1. The light source is not complete linear polarization [10];
2. Missing alignment of the optical setup [11]^,^;
3. Using imperfect optical components, such as, the poor extinction ratio of a polarizer or polarization beam-splitter [12].

Suppose that the polarizer or polarization beam-splitter is imperfect, it can be found that a small amount of the TE state exists in the TM arm and vice versa. Thus, the electric fields of the TM and TE arms are given by, respectively [10,12–13]:

(20) $$E_{\mathrm{TM}}=\left({\mathrm{Ae}}^{i\omega t/2}+{\alpha e}^{-i\omega t/2}\right)e^{i\omega_{0}t}$$

(21) $$E_{\mathrm{TE}}=\left({\mathrm{Be}}^{-i\omega t/2}+{\mathrm{\beta e}}^{i\omega t/2}\right)e^{i\omega_{0}t},$$

where *ω* and *ω*_0_ are the angular frequencies of heterodyne light and optical light source, respectively; *A* = |*A*|*e*^*iϕ*_*A*_^ and *B* = |*B*|*e*^*iϕ*_*B*_^ are the amplitudes of p- and s-polarizations, respectively; and *α* = |*α*|*e*^*iϕ*_*α*_^ and *β* = |*β*|*e*^*iβ*_*β*_^ are the polarization mixing amplitudes of s- and p-polarizations, respectively.

If the transmission axis of analyzer with respect to x-axis is equal to 45° and all phase are the same, such as *ϕ_α_ = ϕ_A_*, *ϕ_β_* = *ϕ_B_* and *ϕ_A_* = *ϕ_B_*, then the reference beam signal *I_r_* is given by [14]:

(22) $$I_{r}={\left|E_{r}\right|}^{2}=\frac{1}{2}\left[{\left({\left|A\right|}^{2}+\left|B\right|\right)}^{2}+{\left({\left|\alpha \right|}^{2}+\left|\beta \right|\right)}^{2}+2\left(\left|A\right|\left|\alpha \right|+\left|B\right|\left|\beta \right|+\left|A\right|\left|B\right|+\left|B\right|\left|\beta \right|\right)\cos\omega t\right]$$

Similarly, the test beam signal *I_t_* is:

(23) $$\begin{array}{ll} I_{t} & ={\left|E_{t}\right|}^{2}\\ & =\frac{1}{2}[{\left|A\right|}^{2}+{\left|B\right|}^{2}+{\left|\alpha \right|}^{2}+{\left|\beta \right|}^{2}+2\left(\left|A\right|\left|\alpha \right|+\left|B\right|\left|\beta \right|\right)\text{cos}\;\omega t+2\left(\left|A\right|\left|\beta \right|+\left|B\right|\left|\alpha \right|\right)\text{cos}\;\phi \\ & \;\;\;\;\;\;\;+2\left|A\right|\left|B\right|\cos\left(\omega t+\phi \right)+2\left|\alpha \right|\left|\beta \right|\cos\left(\omega t-\phi \right)] \end{array}$$

(24) $$\begin{array}{l} ={\left|A\right|}^{2}+{\left|B\right|}^{2}+{\left|\alpha \right|}^{2}+{\left|\beta \right|}^{2}+2\left(\left|A\left|\beta \right|+\left|B\right|\left|\alpha \right|\right|\right)\cos\;\phi \\ \;\;\;+2\sqrt{{\left(\left|A\right|\left|\alpha \right|+\left|B\right|\left|\beta \right|+\left(\left|A\right|\left|B\right|+\left|\alpha \right|\left|\beta \right|\right)\cos\phi \right)}^{2}+{\left(\left|A\right|\left|B\right|+\left|\alpha \right|\left|\beta \right|\right)}^{2}\;\sin^{2}\;\phi }\cos(\omega t+\phi \prime ), \end{array}$$

where *ϕ′* and *ϕ* are the measured value and the theoretical value, respectively. And *ϕ′* is given by:

(25) $$\phi^{\prime }=\tan^{-1}\left[\frac{\left(\left|A\right|\left|B\right|-\left|\alpha \right|\left|\beta \right|\right)\sin\;\phi }{\left(\left|A\right|\left|\alpha \right|+\left|B\right|\left|\beta \right|\right)+\left(\left|A\right|\left|B\right|+\left|\alpha \right|\left|\beta \right|\right)\cos\phi }\right].$$

**Figure 9.**
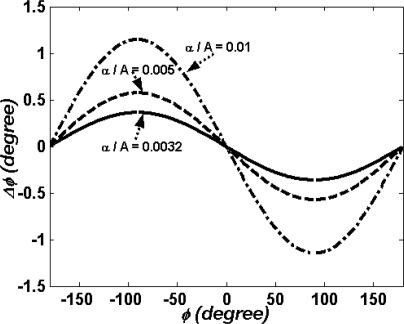
The first order error as a function of the phase difference *ϕ*.

Thus, the first order error due to polarization mixing is Δ*ϕ_m_* = *ϕ′* − *ϕ*. For convenience, we set |*A*| = |*B*| and |*α*| = |*β*|. Figure 9 shows the first order error as a function of the phase difference *ϕ*. It is evident that the maximum error is 0.36° if the extinction ratio is equal to 1 × 10^−5^, i.e. |α||A|=0.0032.

### (b). Second Harmonic Error

**Figure 10.**
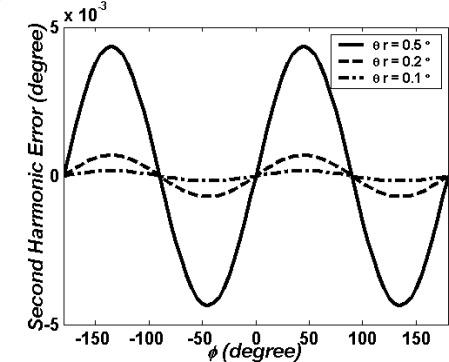
The second harmonic error Δ*ϕ_r_* as a function of the phase difference *ϕ* for different polarization rotation angles.

Second harmonic error is due to the polarization rotation between s- and p-polarizations. And the second harmonic error Δ*ϕ_r_* is given by [15]:

(26) $${\Delta \phi }_{r}={\text{tan}}^{-1}\left(\frac{\text{sin}\;\phi }{\text{cos}\;{2\theta }_{r}\;\text{cos}\;\phi }\right)-\phi ,$$

where *θ_r_* is the polarization rotation angle. As shown in Figure 10, the second harmonic error Δ*θ_r_* is a function of the phase difference *ϕ* for different polarization rotation angles. It is clear that the second harmonic error Δ*θ_r_* is close to zero if the polarization rotation angle *θ_r_* = 0.1°.

Besides, if the phase difference error only results from the resolution of the lock-in amplifier, the phase difference error Δ*ϕ* is given by:

(27) $$\Delta \phi =\frac{d\left({\Delta \phi }_{t}\right)}{dz}\Delta z=\frac{d\left({\Delta \phi }_{t1}+{\Delta \phi }_{t2}\right)}{dz}\Delta z.$$

Because the resolution Δ*ϕ* of the lock-in amplifier is equal to 0.01°, then the small-displacement measurement error *Δz,* of the system can be obtained by:

(28) $${\Delta z}_{r}=\Delta z=\frac{\Delta \phi }{d({\Delta \phi }_{t1}+{\Delta \phi }_{t2})/\mathrm{dz}}.$$

**Figure 11.**
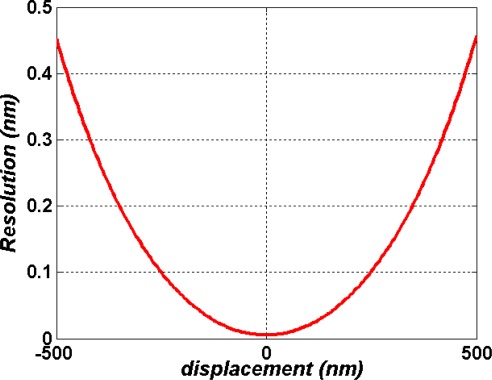
The theoretical displacement resolution of the small-displacement sensor.

As a matter of fact, the *Δz* can be regarded as the solution of the system if the inherent nonlinear errors have been adjusted to zero by choosing high performance optical components and aligning the optical system carefully. According to Equation (28), the theoretical displacement resolution of the small-displacement sensor can reach 0.45 nm in the displacement range of −500 nm ≤ *Δz* ≤ +500 nm, as shown in Figure 11.

## 5. Conclusions

In the paper, a small displacement can be measured by simply measuring the variation in phase difference between s- and p-polarization states with the small-displacement sensor. Besides, the optical structure is designed as a common-path structure and the principle is based on the TIR theory and SPR technology in heterodyne interferomery. Thus, it is stable against the turbulences of the environment such as air turbulences or mechanical vibrations. The new instrument has some distinct advantages: e.g., high resolution, high sensitivity, rapid measurement.

## References

[1] Nesci A., Dändliker R., Herzig H.P. (2001). Quantitative amplitude and phase measurement by use of a heterodyne scanning near-field optical microscope. Opt. Lett.

[2] Liu X., Clegg W., Jenkins D.F.L., Liu B. (2001). Polarization interferometer for measuring small displacement. IEEE Trans. Instrum. Meas.

[3] Liao S.J., Wang S.F., Chiu M.H. (2005). A new method for measuring a small displacement by using the critical angle method and confocal technology. SPIE.

[4] Chiu M.H., Shih B.Y., Lai C.W., Shyu L.H., Wu T.H. (2008). Small absolute distance measurement with nanometer resolution using geometrical optics principles and a SPR angular sensor. Sens. Act. A.

[5] Born M., Wolf E. (1980). Principles of Optics.

[6] Kretshmann E. (1971). Die Bestimmung optischer Konstanten von Metallen durch Anregung von Oberflächenplasmaschwingungen. Z. Phys.

[7] Raether H. (1988). Surface plasmons on smooth and rough surfaces and on gratings.

[8] Wang S.F., Chiu M.H, Lai C.W., Chang R.S. (2006). High-sensitivity small-angle sensor based on the SPR technology and heterodyne interferometry. Applied Optics.

[9] Chiu M.H., Wang S.F., Chang R.S. (2004). Instrument for measuring small angles by use of multiple total internal reflections in heterodyne interferometry. Applied Optics.

[10] Hou W., Wilkening G. (1992). Investigation and compensation of the nonlinearity of heterodyne interferometers. Prec.Eng.

[11] Bobroff N. (1993). Recent advances in displacement measuring interferometry. Meas. Sci. Technol.

[12] Hou W., Zhao X. (1994). Drift of nonlinearity in the heterodyne interferometer. Prec. Eng.

[13] Bobroff N. (1987). Residual errors in laser interferometry fromair turbulence and nonlinearity. Applied Optics.

[14] Badami V.G., Patterson S.R. (2000). A frequency domain method for the measurement of nonlinearity in heterodyne interferometry. Prec.Eng.

[15] Freitas J.M.D., Player M.A. (1993). Importance of rotational beam alignment in the generation of second harmonic errors in laser heterodyne interferometry. Meas. Sci. Technol.

